# Supplementary material for: Data sharing upon request and statistical consistency errors in psychology: A replication of Wicherts, Bakker and Molenaar (2011)
Source: PLoS One. 2023 Apr 13;18(4):e0284243. doi: 10.1371/journal.pone.0284243 (PMC10101414; doi:10.1371/journal.pone.0284243)
Supplement: S1 File — (PDF) [file pone.0284243.s001.pdf]

Supplemental Material

Aline Claesen Wolf Vanpaemel Anne-Sofie Maerten Thomas Verliefde
Francis Tuerlinckx Tom Heyman

Contents

1 Original results reported in Wicherts et al. [1] 1

2 Retracted manuscripts 1

3 Robust variance estimator 4

4 Discarded multiverse analyses 4

5 False positives: Comma versus decimal point as delimiter in decimal numbers 4

6 Duplicate triplets within papers 6

7 Full regression outputs from discussion 6

7.1 What if Wicherts and colleagues had applied *statcheck*? 7

7.2 Why do results with the manual check and *statcheck* differ? 7

References 8

1 Original results reported in Wicherts et al. [1]

Table 1 shows the original output of the negative binomial regression of data sharing on consistency errors, as reported in Table 2 in Wicherts et al. [1].

Table 1: Original output of the negative binomial regression on consistency errors as reported in Table 2 in the paper by Wicherts et al.

	$\hat{\beta}$	SE	Wald $\chi^2_{df=1}$	p
intercept	-2.76	1.30	4.53	0.033
shared	-0.83	0.38	4.84	0.028
$\sqrt{p'}$	4.39	6.13	0.51	0.473
ln(#statistics)	0.85	0.41	4.19	0.041
$\hat{k}$	0.83	0.46		

2 Retracted manuscripts

We searched for retractions in the original sample from Wicherts et al. [2] and in our sample from Vanpaemel et al. [3]. Table 2 includes the search terms and results. We found one retracted paper in the sample from Wicherts et al. [2]: *Method matters: Effects of explicit versus implicit social comparisons on activation,*

behavior, and self-views by Diederik Stapel and Jerry Suls [4]. We did not find retractions in the sample from Vanpaemel et al. [3].

Table 2: Search terms and results for retracted manuscripts

database	search terms	hits
**JPSP**		
Web of Science	(IS = 0022-3514 AND PY = 2004) AND LANGUAGE: (English) AND DOCUMENT TYPES: (Retraction)	0
Web of Science	(IS = 0022-3514 AND PY = 2004) AND LANGUAGE: (English) AND DOCUMENT TYPES: (Retracted Publication)	0
Scopus	(ISSN (0022-3514) AND TITLE-ABS-KEY (retract*) AND TITLE-ABS-KEY (2004))	1
retraction watch	journal: Journal of Personality and Social Psychology; From Date: 11/01/2004 To: 12/31/2004	1
**jep:lmc**		
Web of Science	(IS = 2329-8456 AND PY = 2004) AND LANGUAGE: (English) AND DOCUMENT TYPES: (Retraction)	0
Web of Science	(IS = 2329-8456 AND PY = 2004) AND LANGUAGE: (English) AND DOCUMENT TYPES: (Retracted Publication)	0
Scopus	(ISSN (0278-7393) AND TITLE-ABS-KEY (retract*) AND TITLE-ABS-KEY (2004))	0
retraction watch	journal: Journal of Experimental Psychology: Learning, Memory, and Cognition; From Date: 11/01/2004 To: 12/31/2004	0
**Emotion**		
Web of Science	(IS = (1528-3542) AND PY = 2012) AND LANGUAGE: (English) AND DOCUMENT TYPES: (Retraction)	0
Web of Science	(IS = (1528-3542) AND PY = 2012) AND LANGUAGE: (English) AND DOCUMENT TYPES: (Retracted Publication)	0
Scopus	(ISSN (1528-3542) AND TITLE-ABS-KEY (retract*) AND TITLE-ABS-KEY (2012))	0
retraction watch	journal: Emotion; From Date: 01/01/2012 To: 12/31/2012	0
**Psychology and Aging**		
Web of Science	(IS = (0882-7974) AND PY = 2012) AND LANGUAGE: (English) AND DOCUMENT TYPES: (Retraction)	0
Web of Science	(IS = (0882-7974) AND PY = 2012) AND LANGUAGE: (English) AND DOCUMENT TYPES: (Retracted Publication)	0
Scopus	(ISSN (0882-7974) AND TITLE-ABS-KEY (retract*) AND TITLE-ABS-KEY (2012))	0
retraction watch	journal: Psychology and Aging; From Date: 01/01/2012 To: 12/31/2012	0
**JAP**		
Web of Science	(IS = (0021-843X) AND PY = 2012) AND LANGUAGE: (English) AND DOCUMENT TYPES: (Retraction)	0
Web of Science	(IS = (0021-843X) AND PY = 2012) AND LANGUAGE: (English) AND DOCUMENT TYPES: (Retracted Publication)	0
Scopus	(ISSN (0021-843x) AND TITLE-ABS-KEY (retract*) AND TITLE-ABS-KEY (2012))	0
retraction watch	journal: Journal of Abnormal Psychology; From Date: 01/01/2012 To: 12/31/2012	0
**ECP**		
Web of Science	(IS = (1064-1297) AND PY = 2012) AND LANGUAGE: (English) AND DOCUMENT TYPES: (Retraction)	0
Web of Science	(IS = (1064-1297) AND PY = 2012) AND LANGUAGE: (English) AND DOCUMENT TYPES: (Retracted Publication)	0
Scopus	(ISSN (1064-1297) AND TITLE-ABS-KEY (retract*) AND TITLE-ABS-KEY (2012))	0
retraction watch	journal: Experimental and Clinical Psychopharmacology; From Date: 01/01/2012 To: 12/31/2012	0

3 Robust variance estimator

Heteroscedasticity is common in time-series, cross sectional, longitudinal and clustered data (see https://rdocumentation.org/packages/sandwich/versions/3.0-1). In this case, a robust variance estimator is indicated, instead of a standard variance estimator. It is not clear which estimator is applied in SPSS 18.0 [5]. Because we do not assume that the observations are ordered, we do not apply an Heteroscedasticity and Autocorrelation Consistent (HAC) Covariance Matrix Estimation (vcovHAC, the estimator in the Zelig package), but an Heteroscedasticity-Consistent Covariance Matrix Estimation (vcovHC) [6]. See Table 3 for standard error estimates of the coefficients of the negative binomial regression in SPSS, the original *Zelig* output (version unknown), the reproduction *Zelig* output (version 4.2.1) [7], and the reproduction with *sandwich* (version 3.0.1) [8]. The estimations vary a bit, but vcovHC (type HC0) gives the smallest estimates.

Table 3: Standard error estimations in different software (packages) and versions

	coefficient	SPSS	Zelig original	Zelig reproduction	sandwich
intercept	-2.762	1.297	1.267	1.251	1.190
shared	-0.830	0.377	0.391	0.391	0.365
$\sqrt{p'}$	4.394	6.128	6.808	6.870	6.422
ln(# statistics)	0.848	0.414	0.362	0.359	0.341

4 Discarded multiverse analyses

We include these initial multiverse analyses here for sake of transparency, but the results should be interpreted with extra caution. The following count models were fit:

- Poisson regression
- quasipoisson regression
- *negative binomial regression*
- zeroinflated poisson regression
- zeroinflated negative binomial regression

The combinations of covariates were equivalent to the ones in the main text for each count model. In the zeroinflated models, the zero part consisted of all combinations with the square root of the mean of recalculated p-values, the journal in which the paper is published, and the natural logarithm of the number of statistics. For the Poisson, quasipoisson and negative binomial regressions, we also estimated the standard errors of the regression coefficients with a robust variance estimator. The models were fit on the original data (see Fig 1) and on replication data with uncorrected evaluations of consistency errors (see Fig 2), we did not include alternative data set constructions. In total, 17 models (all zeroinflated negative binomial regressions) returned computational warnings. In the original data, 125 out of the 136 model specifications, returned a p-value smaller than or equal to .05. In the replication data, there was a completely different distribution of p-values, where 15 out of the 136 model specifications returned a p-value smaller than or equal to .05. We decided to drop all zeroinflated models, because of the computational warning. Further, we also decided to leave out the Poisson and quasipoisson regressions, because the negative binomial regressions were more appropriate and provided a better fit.

5 False positives: Comma versus decimal point as delimiter in decimal numbers

In the *statcheck* results, we found three test statistic that were reported with a comma instead of a decimal point as delimiter.

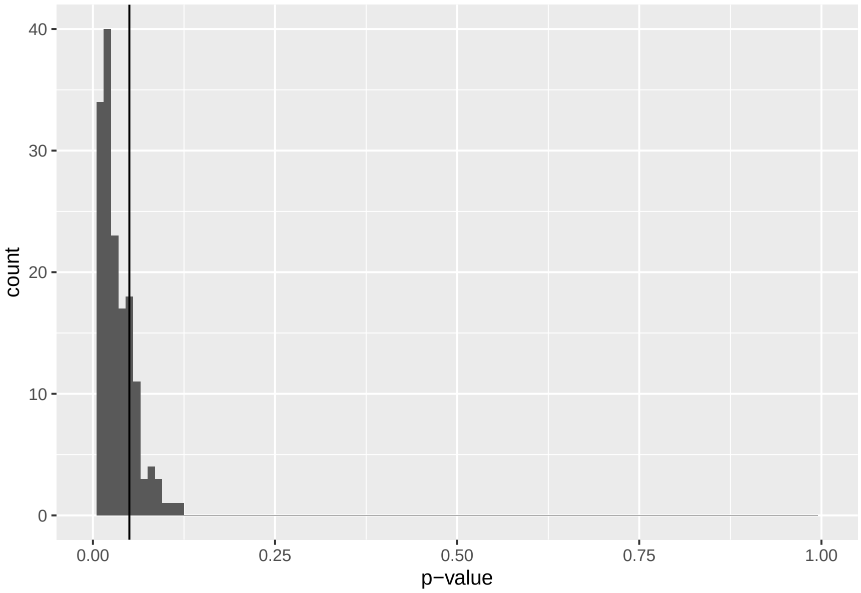

Figure 1: Histogram of p-values from initial multiverse analysis on the original data

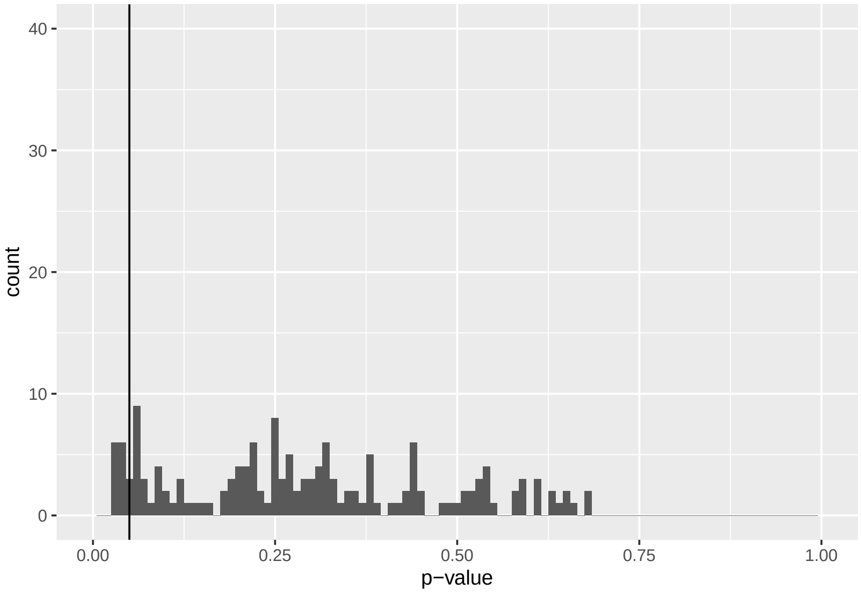

Figure 2: Histogram of p-values from initial multiverse analysis on the replication data

- $F(1,77) = 12,19, p = .001$ was read by *statcheck* as $F(1,77) = 1219, p = .001$, while it should be read as $F(1,77) = 12.19, p = .001$.
- $t(43) = -1,61, p = .12$ was read by *statcheck* as $t(43) = -161, p = .12$, while it should be read as $t(43) = -1.61, p = .12$
- $F(1,40) = 130,49, p < .01$ was read by *statcheck* as $F(1,40) = 13049, p < .01$, while it should be read as $F(1,40) = 130.49, p < .01$

In the *statcheck* output, we corrected the columns `Value` (the value of the test statistic) and `Computed` (the recalculated p-value).

For other kinds of false positives, we did not correct any columns of the *statcheck* results.

6 Duplicate triplets within papers

Duplicate triplets within papers can occur for several reasons. First of all, *statcheck* reads the entire paper and not only the results section. Interesting results can be repeated in the abstract, discussion, footnote or figure caption, and thereby read multiple times by statcheck. Second, it's possible that the authors found the same result more than once. Finally, when authors have to publish many similar results, they might copy-paste one result and use it as a template for the other results. However, if they forget to adapt the copied result, statcheck will read the result multiple times. A quick look at the papers with duplicated results learned us that it is difficult to retrieve the cause of the duplicates. As we do not know how [1] decided to include or exclude duplicate statistics, we removed all duplicate statistics in the main replication analysis. Here, we repeat the main replication analysis with duplicate statistics, because they are not necessarily the repetition of an interesting result or a copy-paste error. Table 4 and 5 show that the replication results with duplicate triplets do not differ much from the replication results reported in the main text. In fact, 5 does not differ at all.

Table 4: Replication of the relationship between data sharing and consistency errors (without excluding duplicate statistics)

	$\hat{\beta}$	SE	z	p	2.5%	97.5%
intercept	-3.58	0.44	-8.11	$< .001$	-4.44	-2.71
shared	-0.17	0.23	-0.73	0.46	-0.62	0.28
$\sqrt{p'}$	12.85	3.09	4.16	$< .001$	6.79	18.9
ln(#statistics)	0.88	0.12	7.54	$< .001$	0.65	1.11
$\hat{\theta}$	0.82	0.18				
AIC	586.94					

Table 5: Data sharing and presence of decision errors per paper (without excluding duplicate statistics)

	no decision errors	at least one decision error
not shared	164	14
shared	101	7

7 Full regression outputs from discussion

This section includes the full regression outputs of the results in the discussion on whether statcheck is a good proxy for the manual check for consistency errors conducted by [1].

7.1 What if Wicherts and colleagues had applied *statcheck*?

Table 6 reports the results for the analysis on the consistency errors as detected by statcheck in default mode. Table 7 shows the results when consistency errors were detected by statcheck with automatic one-tail detection.

Table 6: Results when consistency errors were detected by statcheck without automatic one-tail detection

	$\hat{\beta}$	SE	z	p	2.5%	97.5%
intercept	-2.08	0.78	-2.68	0.01	-3.60	-0.56
shared	0.25	0.50	0.51	0.61	-0.72	1.23
$\sqrt{p'}$	6.31	5.67	1.11	0.27	-4.81	17.43
ln(#statistics)	0.66	0.29	2.29	0.02	0.09	1.22
$\hat{\theta}$	0.81	0.33				
AIC	150.96					

Table 7: Results when consistency errors were detected by statcheck with automatic one-tail detection

	$\hat{\beta}$	SE	z	p	2.5%	97.5%
intercept	-2.20	0.80	-2.75	0.01	-3.76	-0.63
shared	0.45	0.54	0.83	0.40	-0.61	1.52
$\sqrt{p'}$	3.52	5.93	0.59	0.55	-8.11	15.14
ln(# statistics)	0.68	0.30	2.31	0.02	0.10	1.26
$\hat{\theta}$	0.78	0.34				
AIC	136.90					

7.2 Why do results with the manual check and *statcheck* differ?

The results of the analysis on statistics extracted by both statcheck and the manual check done by Wicherts et al. [1] can be found in Table 8 if consistency errors were evaluated by Wicherts et al., in Table 9 if consistency errors were evaluated by default statcheck, and Table 10 if consistency errors were evaluated by statcheck with automatic one-tail detection.

Table 8: Results on intersection of statistics, evaluated manually

	$\hat{\beta}$	SE	z	p	2.5%	97.5%
intercept	-1.63	0.71	-2.31	0.02	-3.01	-0.24
shared	-0.77	0.40	-1.93	0.05	-1.56	0.01
$\sqrt{p'}$	-0.86	6.78	-0.13	0.90	-14.15	12.43
ln(#statistics)	0.66	0.32	2.11	0.03	0.05	1.28
$\hat{\theta}$	1.53	1.13				
AIC	111.16					

Table 9: intersection with default statcheck

	$\hat{\beta}$	SE	z	p	2.5%	97.5%
intercept	-2.01	0.85	-2.35	0.02	-3.68	-0.33
shared	0.41	0.52	0.78	0.43	-0.61	1.42
$\sqrt{p'}$	5.88	5.87	1.00	0.32	-5.63	17.38
ln(#statistics)	0.61	0.34	1.78	0.08	-0.06	1.27
$\hat{\theta}$	0.74	0.31				
AIC	141.62					

Table 10: intersection with automatic one-tailed detection

	$\hat{\beta}$	SE	z	p	2.5%	97.5%
intercept	-2.14	0.91	-2.34	0.02	-3.94	-0.35
shared	0.65	0.58	1.12	0.26	-0.49	1.79
$\sqrt{p'}$	3.09	6.24	0.49	0.62	-9.14	15.31
ln(#statistics)	0.62	0.36	1.70	0.09	-0.10	1.33
$\hat{\theta}$	0.75	0.35				
AIC	126.30					

References

1. Wicherts JM, Bakker M, Molenaar D. Willingness to Share Research Data Is Related to the Strength of the Evidence and the Quality of Reporting of Statistical Results. Tractenberg RE, editor. PLoS ONE. 2011;6: e26828. doi:10.1371/journal.pone.0026828
2. Wicherts JM, Borsboom D, Kats J, Molenaar D. The poor availability of psychological research data for reanalysis. American Psychologist. 2006;61: 726–728. doi:10.1037/0003-066X.61.7.726
3. Vanpaemel W, Vermorgen M, Deriemaecker L, Storms G. Are We Wasting a Good Crisis? The Availability of Psychological Research Data after the Storm. Collabra. 2015;1: 1–5. doi:10.1525/collabra.13
4. Retraction to Method matters: Effects of explicit versus implicit social comparisons on activation, behavior, and self-views [Journal of Personality and Social Psychology, 87 (2004) 860-875] doi: 10.1037/0022-3514.87.6.860. 2013. p. 163. doi:10.1037/a0031425
5. SPSS Inc. PASW Statistics for Windows/Macintosh. Chicago: SPSS Inc; 2009.
6. Zeileis A. Econometric Computing with HC and HAC Covariance Matrix Estimators. Journal of Statistical Software. 2004;11: 1–17. doi:10.18637/jss.v011.i10
7. Owen M, Imai K, King G, Lau O. Zelig: Everyone's statistical software [Internet]. 2013. Available: http://gking.harvard.edu/zelig
8. Zeileis A, Lumley T. Sandwich: Robust covariance matrix estimators [Internet]. 2021. Available: https://sandwich.R-Forge.R-project.org/
