# Supplementary material for: Data sharing upon request and statistical consistency errors in psychology: A replication of Wicherts, Bakker and Molenaar (2011)
Source: PLoS One. 2023 Apr 13;18(4):e0284243. doi: 10.1371/journal.pone.0284243 (PMC10101414; doi:10.1371/journal.pone.0284243)
Supplement: Attachment — Submitted filename: CB_pone-d-22-16726_review.rtf [file pone.0284243.s002.rtf]

PONE-D-22-16736 

Thank you for the opportunity to review this manuscript. Replication studies, especially those regarding issues with data sharing, are both timely and important. The manuscript was well-written and replication was comprehensive while being transparent. Overall, the manuscript would benefit from minimal revisions mainly aiming at contextualizing the impact of the study. 

1. One reason Wicherts et al. (2011) conducted their study is because it is an ethical imparative for psychologists to share their data upon request by other psychologists. Noting the importance of why authors' reluctance to share data and syntax/code for verifying results would strengthen the impact of the manuscript. From an ethics standpoint, Bosma & Granger (2022) provide a recent commentary on the ethical implications related to data sharing, which could be helpful for contextualizing why this manuscript is important. 

Bosma, C. M., & Granger, A. M. (2022). Sharing is caring: Ethical implications of transparent research in psychology.  American Psychologist. 

2. Although it is noted in the discussion that Wicherts et al. results did not replicate with the new sample, more discussion can be provided on considering potential cohort effects. Awareness of open data practices have been growing over the past ~15 years and researchers may be more careful about reporting results since Wicherts et al and others began shedding more light on this issue. Though this cannot be tested given the data available to the authors, it is worth discussing. 

3. More discussion should be provided regarding sampling bias. Transparency in research varies greatly between sub-disciplines in psychology, yet the the authors attempted replicating Wicherts et al. using convenient data from different journals. Further, although APA journals fall under one organization, the policies for each journal differ greatly in addition to sub-discipline norms. Greater acknowledgement of the different journals included in the replication study would be helpful to further provide context for the degree of inference that can be made from the replication results. 

4. Building on comment 1, the authors can add several statements in the introduction and conclusion for why the study broadly matters. Psychologists are ethically supposed to share their data for verification as part of ensuring that what is reported is accurate. The bigger story is that psychologists are still reluctant to share their data upon request, but is helpful to know that perhaps reluctance to sharing is not clearly connected with consistency/decision errors.
